# Sedentary Leisure Behaviour, Physical Activity, and Gastroesophageal Reflux Disease: Evidence From a Mendelian Randomization Analysis

**DOI:** 10.1002/hsr2.70479

**Published:** 2025-03-02

**Authors:** Shan Lu, Yahong Zhu, Mingyu Cui, Zheng Guo, Xingang Li, Ying Song

**Affiliations:** ^1^ Gastroenteric Medicine and Digestive Endoscopy Center, The Second Hospital of Jilin University Changchun Jilin China; ^2^ School of Medical and Health Sciences Edith Cowan University Joondalup Western Australia Australia; ^3^ School of Sciences Edith Cowan University Joondalup Western Australia Australia; ^4^ Centre for Precision Health Edith Cowan University Joondalup Western Australia Australia

**Keywords:** causal effect, gastroesophageal reflux disease, leisure sedentary behaviours, Mendelian randomization, physical activities

## Abstract

**Background and Aims:**

Gastroesophageal reflux disease (GERD) is common worldwide. Although associations between sedentary behaviour (LSBs), physical activity (PAs), and GERD have been reported, their causal relationships remain unclear. This study uses Mendelian randomization (MR) to examine these associations with GERD.

**Methods:**

Genetic instruments from the UK Biobank and other consortia were utilized to assess the causal relationship between LSBs, PAs, and GERD. Causal analyses employed inverse variance weighted (IVW), weighted‐median (WM), and MR‐Egger regression. Sensitivity analyses included Cochran's *Q* test, MR‐Egger intercept test, leave‐one‐out analysis, and funnel plot assessment. Outliers were detected using MR‐PRESSO and Radial MR. A risk factor analysis explored potential causal links.

**Results:**

Genetically predicted leisure television (TV) watching (OR = 2.29, 95% CI 2.12–2.48; *p* < 0.001) and self‐reported moderate physical activity (MPAs) (OR = 2.45, 95% CI 1.46–4.13; *p* < 0.001) were associated with an higher risk of GERD. In contrast, leisure computer use (OR = 0.62, 95% CI 0.53–0.73; *p* < 0.001) and accelerometer‐measured fractional accelerations > 425 milligravities (OR = 0.68, 95% CI 0.52–0.88; *p* = 0.003) were associated with a lower risk of GERD. No causal relationship was observed between driving, self‐reported vigorous physical activities (VPAs), and accelerometer‐measured “average acceleration” with GERD. Risk factor analyses suggested that metabolic risk factors, such as smoking, high body mass index, elevated serum triglyceride levels, insulin resistance, and type 2 diabetes might mediate the observed causal links.

**Conclusions:**

Leisure TV watching and self‐reported MPA are associated with an higher risk of GERD, whereas leisure computer use and accelerometer‐measured fractional acceleration > 425 milligravities may serve as protective factors against GERD. These findings highlight the necessity of differentiating various LSBs and PAs in GERD research.

## 1 Introduction

Gastroesophageal reflux disease (GERD) is a chronic, recurrent disorder characterized by symptoms such as heart burning and acid reflux into the oesophagus. It is a significant health concern in Europe, affecting a substantial portion of the population [1]. GERD manifests in various phenotypes, including non‐erosive reflux disease, esophagitis (low or high‐grade), GERD hypersensitivity, laryngopharyngeal reflux, reflux chest pain, and Barrett's esophagus [2]. Proton pump inhibitors are the primary treatment for GERD. However, multiple symptoms, drug side effects, and poor patient compliance make GERD management challenging [3].

The economic impact of GERD is considerable, with conservative estimates indicating annual healthcare costs and productivity losses of $760 million and $2.4 billion in the UK and the US, respectively [4, 5]. The direct medical costs for GERD patients have increased by $8.6 million. In response, significant efforts have been made to alleviate the overall economic burden associated with GERD treatment and hospitalization [6, 7]. The burden of GERD extends beyond the health care system, significantly affecting patients' quality of life and imposing a strain on national economies. Given these economic and health burdens, understanding GERD's modifiable risk factors is crucial. Therefore, identifying potential risk factors closely associated with GERD is crucial for developing effective prevention and treatment strategies [8, 9].

Epidemiological evidence suggests that several factors, such as poor diet, aging, drug use, and organic lesions, can contribute to the damage to the lower oesophageal sphincter (LES), leading to GERD development. Although physical activities (PAs) are generally beneficial for health, their impact on GERD remains controversial [10, 11]. Some studies have found that PAs can increase acid exposure and the duration and frequency of reflux episodes, indicating a potential association between PAs and GERD exacerbation. In addition, a recent study has also found that exercising 1–3 times per week increases the risk of developing GERD [12, 13, 14]. Conversely, other studies support the view that both intense PAs and moderate daily PAs may have long‐term benefits in preventing GERD [15, 16]. Sedentary behaviours, including activities such as watching TV, and computer use, have been linked to the development of chronic conditions with recurrent episodes [17, 18, 19]. However, the impact of PAs and sedentary behaviours on GERD is still a topic of debate.

Mendelian randomization (MR) studies use genetic instruments to infer causality in clinical diseases [20]. As a novel epidemiological approach, MR studies can establish relationships between phenotypes (exposures) and diseases (outcomes) through genotypes (instrumental variables [IVs]) [21]. MR studies overcome limitations of traditional observational studies by reducing confounding and reverse causality. Unlike randomized controlled trials (RCTs), MR studies do not have ethical and logistical constraints [22]. This is achieved by utilizing genetic IVs that are randomly assigned at conception, long before the onset of the disease [23].

This study aims to evaluate the causal effects of leisure sedentary behaviours (LSBs) (TV watching and computer use) and PAs (moderate and vigorous) on GERD risk using genetic data from the UK Biobank and other consortia. We conducted various sensitivity analyses, including Cochran's *Q* test, MR‐Egger regression, leave‐one‐out (LOO) analysis, and funnel plot. To ensure robustness and address potential pleiotropy, we conducted multiple sensitivity analyses. We employed MR‐Pleiotropy Residual Sum and Outlier (MR‐PRESSO) and Radial MR to identify and address potential pleiotropy. Furthermore, we investigated potential metabolic risk factors that may mediate the observed causal links between LSBs, PAs, and GERD using a risk factor analysis.

## 2 Materials and Methods

### 2.1 Study Design

This MR study was designed to conduct a two‐sample MR analysis utilizing summarized statistics from genome‐wide association study (GWAS) data to examine the causal effects of LSBs and PAs on the GERD susceptibility. This IV analysis mimicked an RCT by leveraging the random allocation of single nucleotide polymorphisms (SNPs) in offspring, independent of confounding factors such as sex and age. To ensure the validity of the MR analysis, the genetic IVs used in this study adhered to three core assumptions: (i) the genetic IVs should exhibit a strong correlation with the exposure factors (*P* < 5 × 10^–8^), (ii) the genetic IVs need to be independent of potential confounders, and (iii) the exposure factors represent the sole pathway through which the genetic IVs influence the outcome [20, 24].

### 2.2 GWAS Summary Data for Exposures

The candidate genetic instruments for LSBs were derived from a recently published summary‐level GWAS, which consisted of a sample of 422,218 participants predominantly of European ancestry from the UK Biobank [18]. The GWAS meta‐analysis identified three types of LSBs: leisure TV watching, leisure computer use, and driving. The researchers assessed the duration of each sedentary behaviour by asking the participants questions such as “On a typical day, how many hours do you spend watching TV?”, “On a typical day, how many hours do you spend using a computer (excluding computer use at work)?”, and “On a typical day, how many hours do you spend driving?”. Previous reports indicated that the average daily time spent on TV watching was 2.8 hours (h) (standard deviation [SD] 1.5 h), computer use was 1.0 h (SD 1.2 h), and driving was 0.9 h (SD 1.0 h). Adjustments were made for various factors, including age, gender, body mass index (BMI), smoking status, hypertension, diabetes, Townsend deprivation index, PA levels, alcohol consumption per week, and years of education in the analysis. Further information regarding the data can be found in the published GWAS meta‐analysis [18].

The summarized GWAS data for self‐reported and accelerometry‐based PAs were obtained from a large prospective study that recruited nearly 500,000 adults aged 40 to 69 from various ethnic backgrounds at multiple UK Biobank centers between 2006 and 2010 [25]. Four PAs phenotypes were selected, including self‐reported moderate PAs (MPAs) (*n* = 343,827), self‐reported vigorous PAs (VPAs) (*n* = 261,055), accelerometer‐measured “average acceleration” (*n* = 91,084), and accelerometer‐measured fraction accelerations > 425 milligravities (*n* = 90,667). In the UK Biobank, self‐reported PA data were obtained by volunteers through a touchscreen questionnaire, which was similar to the International Physical Activity Questionnaire (IPAQ) [26]. Objective accelerometer‐measured PA data were collected from 100,000 participants who wore the Axivity AX3 wrist‐worn accelerometer for 7 days [27]. Adjustments were made for various covariates, including age, sex, genotyping chip, first ten genomic principal components, and center. Further information regarding the PA data are available in the published GWAS meta‐analysis [28, 29].

### 2.3 Genetic IVS

To ensure accurate conclusions, we implemented rigorous quality control measures to identify qualified IVs. First, the selected SNPs had to meet the genome‐wide significance threshold (*P* < 5 × 10^−8^). Second, we addressed the potential influence of linkage disequilibrium (LD) by ensuring the independence of the IVs. We used the PLINK method [30] to clump the SNPs (with *r* ^2^ < 0.001 and a genome region size of 10,000 kb). Third, we excluded weak instrumental variables by setting a minimum F‐statistic value of >10 [31]. Fourth, we harmonized the effect sizes and alleles of SNPs between the exposure and outcome data. We also removed erroneous data, including palindromic SNP [with an effect allele frequency (EAF) > 0.42] and SNPs strongly associated with the outcome (with *P* < 5 × 10^−8^). Finally, we employed methods such as MR‐pleiotropic residuals and outliers (MR‐PRESSO) and radial MR to remove the SNPs with potential pleiotropy, and MR Analysis was performed for stability assessment. Since only a very small number of SNPs were lost during these steps, we did not utilize proxy SNPs to replace them, as their impact on the results would be minimal.

**FIGURE 1:**
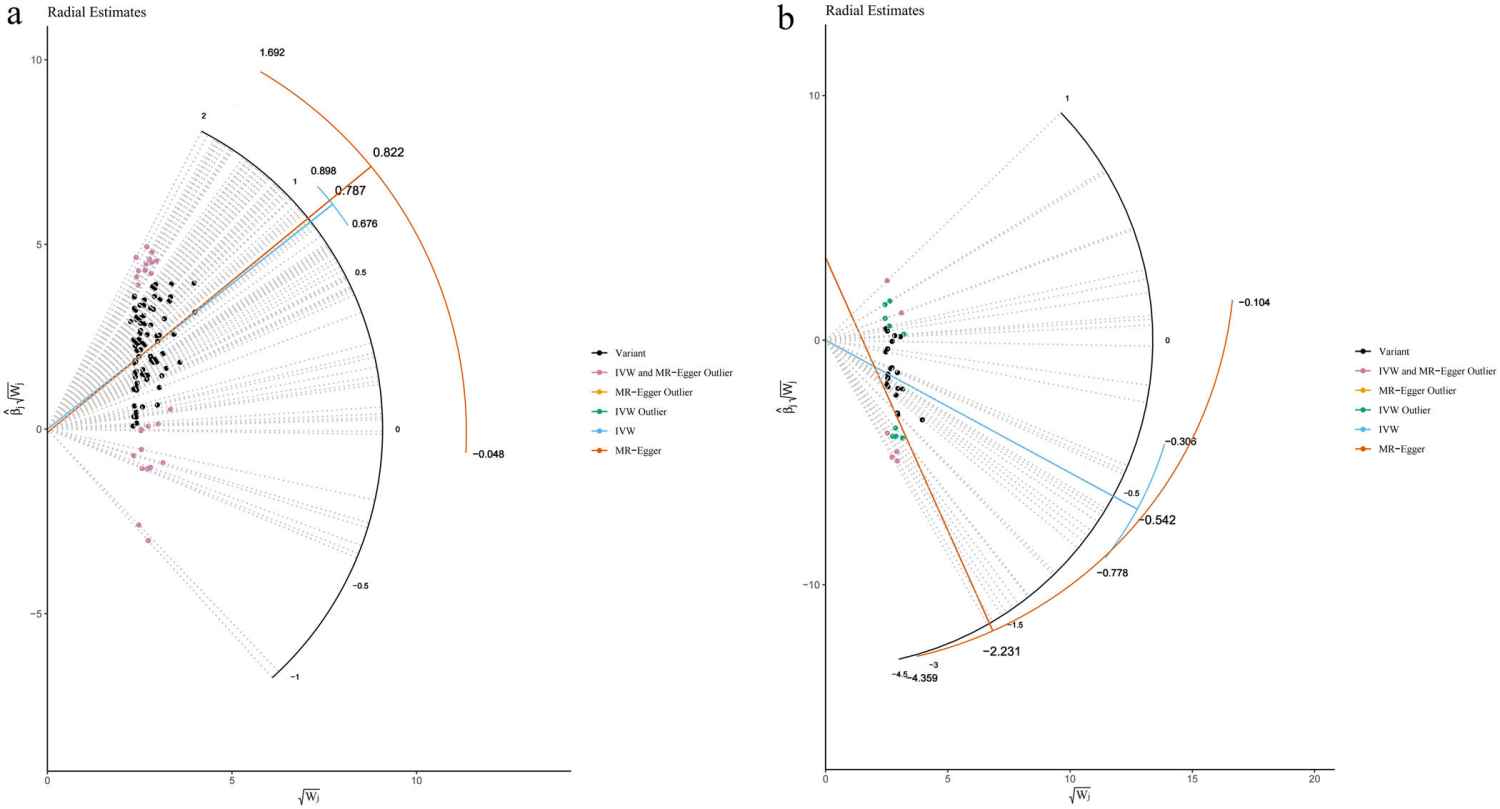
Outliers identified by Radial Mendelian randomization method for TV watching and computer use on GERD (a, b). GERD, gastroesophageal reflux disease; IVW, inverse variance weighted.

The Radial MR estimator was used to identify outliers in TV watching and computer use phenotypes (Figure 1), resulting in the identification and manual removal of 25 and 15 outliers, respectively. Subsequently, 74, 21, and 4 SNPs were utilized as IVs for TV watching, computer use, and driving, respectively. The information about the SNPs selected as IVs was shown in an additional file (Tables S1–S3). To incorporate more SNPs related to accelerometer‐measured fraction accelerations > 425 milligravities, we employed a more lenient threshold (*P* < 5 × 10^−7^), which has been previously used in numerous MR studies [32, 33]. Consequently, our study employed 5, 4, 4, and 4 SNPs for self‐reported MPAs, self‐reported VPAs, accelerometer‐measured “average acceleration” and accelerometer‐measured fraction accelerations > 425 milligravities, respectively. Detailed information regarding these candidate IVs is provided in the additional file (Tables S4–S7).

### 2.4 GWAS Summary Data for Outcome

The GWAS summary statistics for GERD were obtained from a comprehensive GWAS meta‐analysis, which included data from the largest and most recent studies on GERD. This meta‐analysis involved 129,080 European individuals diagnosed with GERD and 473,524 healthy controls. A total of 2,320,781 genetic variations were analyzed in this study [34]. GERD cases were defined based on a combination of self‐reported symptoms, International Classification of Diseases diagnoses, and the use of GERD‐related medications [34]. The aforementioned data can be found in the IEU Open GWAS Project database (https://gwas.mrcieu.ac.uk/datasets/).

All GWAS summarized data included in this study are publicly available. As this study did not involve new data collection or direct interaction with human subjects, separate ethical approval was not required.

### 2.5 Statistical Analysis

The primary analysis in this study focused on the causal relationship between LSBs and PAs with GERD risk, using inverse variance weighted (IVW), MR‐Egger regression, and weighted median (WM) methods as the main approaches. The IVW method, which combines each SNP with the Wald ratio in a meta‐analysis framework, was used to estimate the overall effect of LSBs on GERD. Several complementary methods were used to enhance the robustness of the results under different assumptions. The MR‐Egger regression slope, assuming the instrument strength independent of the direct effect (InSIDE) hypothesis, accurately reflects the causal correlation effect of exposure on the outcome, and a nonsignificant P‐value for the MR‐Egger intercept (>0.05) indicates no directional pleiotropy [35]. The WM method provides accurate effect estimates as long as over 50% of the IVs do not violate the core MR assumptions [36]. Sensitivity analyses, including MR‐PRESSO, radial MR, Cochrane's *Q* test, funnel pot, and MR‐Egger intercept test, were conducted to evaluate the robustness of causal inference. Cochran's *Q* test was used to evaluate statistical heterogeneity among SNPs estimated by IVW. The LOO analysis was employed to examine the impact of each SNP on the pooled estimate, allowing for the detection of potential pleiotropic effects. Additionally, to ensure that the causal inference was not biased by inverse causality, we performed the Steiger test, with a significance level set at *p* < 0.05.

The MR estimates were reported as odds ratios (OR) along with their respective 95% confidence intervals (CIs). These estimates quantified the relative risk of GERD associated with each SD increased the duration of three types of LSBs (leisure TV watching, leisure computer use, and driving) and four types of PAs (self‐reported MPAs, self‐reported VPAs, accelerometer‐measured “average acceleration,” and accelerometer‐measured fraction accelerations >425 milligravities). The primary analysis was based on two‐sided tests, with statistical significance set at *p* < 0.05. Given the multiple exposures and tests, we applied a Bonferroni correction to adjust for multiple comparisons, using a threshold of *p* < 0.007 (0.05/7 exposures). All statistical models were implemented using the *TwoSampleMR* (version 0.5.6) and *MR‐PRESSO* (version 1.0) packages in R software (version 4.1.3).

### 2.6 Risk Factor Analysis

In addition to the primary analysis, exploratory analyses were performed. Due to the possibility of unknown confounders and the absence of specific prior hypotheses. These exploratory analyses aimed to provide further insights into the potential modifiers of the relationships between LSBs, PAs, and GERD. An additional MR analysis was conducted to assess the causal relationship between LSBs and PAs with common risk factors for GERD. Several potential mediators were elected for analysis, including smoking, BMI, type 2 diabetes (T2D), fasting insulin, total cholesterol, triglycerides, insomnia complaints, visceral adipose tissue volume, and years of schooling. Detailed information on these potential mediators is provided in the summary data of the GWAS listed in Table 1.

**TABLE 1:** Data source of the RA‐related risk factors.

Traits	Consortium	Year	Code	Sample size	Ancestry	PubMed ID
Ever versus never smoked	TAG	2010	ieu‐a‐962	74035	European	20418890
Former versus current smoker	TAG	2010	ieu‐a‐963	41969	European	20418890
Cigarettes smoked per day	TAG	2010	ieu‐a‐961	68028	European	20418890
Total cholesterol	GLGC	2013	ieu‐a‐301	187365	Mixed	24097068
Triglycerides	GLGC	2013	ieu‐a‐302	177861	Mixed	24097068
Body mass index	GIANT	2015	ieu‐a‐835	322154	European	25673413
Type 2 diabetes	DIAGRAM	2012	ieu‐a‐26	69033	European	22885922
Fasting insulin	MAGIC	2012	ieu‐b‐115	51750	European	22581228
Years of schooling	SSGAC	2014	ieu‐a‐755	106736	European	30038396
Insomnia complaints	NA	2017	ebi‐a‐GCST004695	113006	European	28604731
Visceral adipose tissue volume	NA	2021	ebi‐a‐GCST90016671	32860	European	34128465

Abbreviations: DIAGRAM, Diabetes Genetics Replication and Meta‐analysis; GIANT, Genetic Investigation of Anthropometric Traits; GLGC, Global Lipids Genetics Consortium; MAGIC, Meta‐Analyses of Glucose and Insulin‐related traits Consortium; SSGAC, Social Science Genetic Association Consortium; TAG, Tobacco and Genetics.

## 3 Results

### 3.1 MR Estimates of the Causal Effects of LSBs on GERD

In the case of the experimental LSBs phenotypes, multiple analyses revealed a significant association between leisure TV watching and an increased risk of GERD. The IVW analysis yielded an odds ratio (OR) of 2.29 (95% confidence interval [CI] 2.12–2.48; *p* < 0.001), indicating a substantial risk. Similarly, the WM analysis yielded an OR of 2.21 (95% CI 1.95–2.49; *p* < 0.001), and the MR‐Egger analysis showed an OR of 2.77 (95% CI 1.72–4.45; *p* < 0.001).

Furthermore, across various MR Methods, a consistent correlation was observed between computer use and GERD. The IVW analysis indicated an OR of 0.62 (95% CI, 0.53–0.73; *p* < 0.001) for each increment of the standard deviation of computer use, suggesting a protective effect against GERD.

**FIGURE 2:**
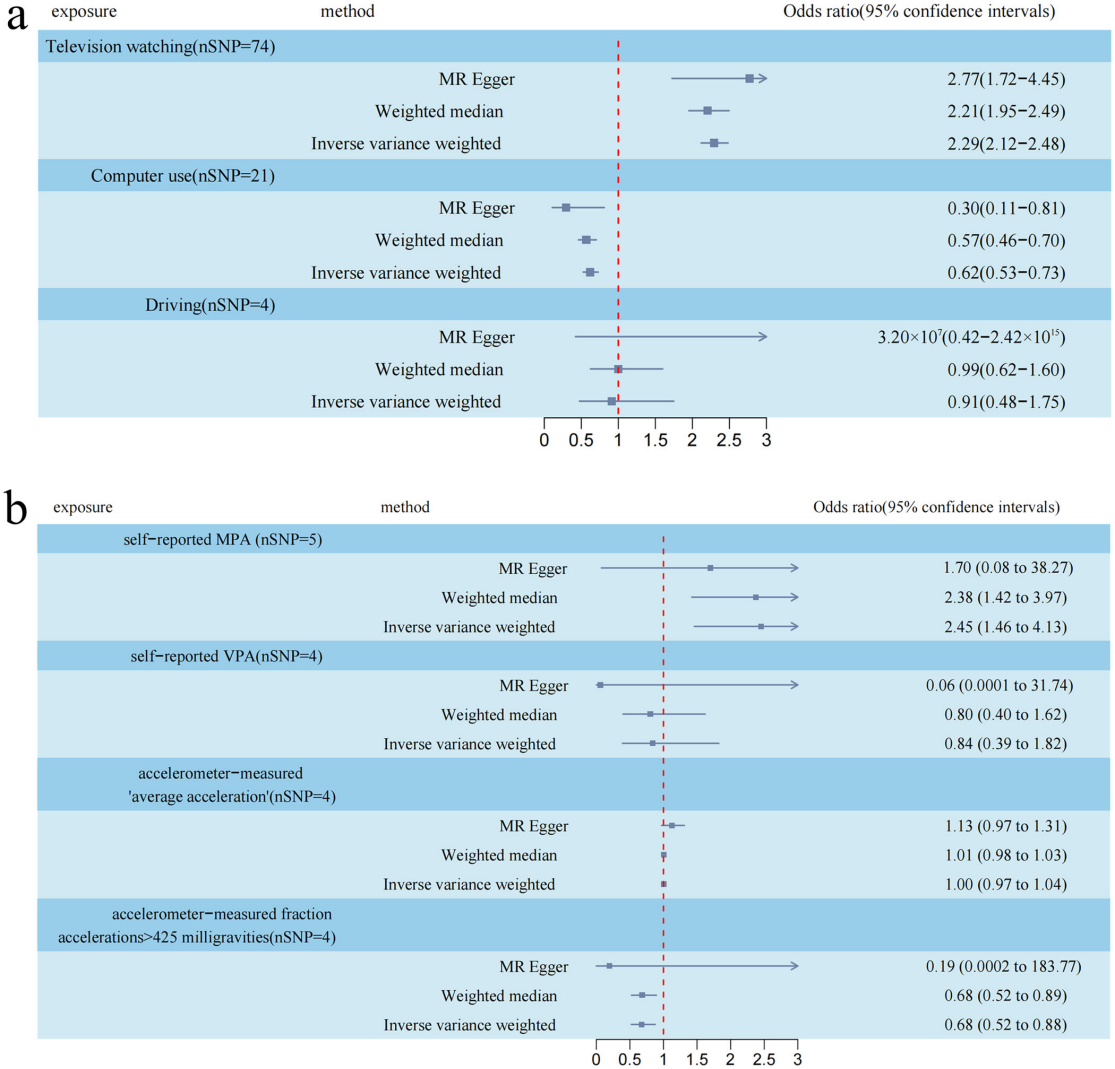
Causal effects for leisure sedentary behaviours and physical activity behaviours on GERD. (a, b) Summary of the Mendelian randomization (MR) estimates derived from the inverse‐variance weighted (IVW), weighted median (WM), MR‐Egger methods. GERD, gastroesophageal reflux disease; MPAs, self‐reported moderate physical activities; VPAs, self‐reported vigorous physical activities.

On the other hand, concerning the driving phenotypes, no observational evidence of causal relationships between genetically predicted LSBs and GERD was found (Figure 2a).

### 3.2 MR Estimates of the Causal Effects of PA on GERD

Among the PA phenotypes tested, the IVW analysis showed that a higher accelerometer‐measured fraction accelerations > 425 milligravities was associated with a reduced risk of GERD (OR = 0.68, 95% CI 0.52–0.88; *p* = 0.003). However, self‐reported moderate PA was found to increase the risk of GERD (OR = 2.45, 95% CI 1.46–4.13; *p* < 0.001). Similar estimates were obtained through the WM analysis. However, the MR Egger's findings suggested a consistent direction of effect, although the magnitude was not readily apparent (Figure 2b). In addition, details on MR estimates of the causal relationships between LSBs/PAs and GERD are shown in Additional file 1: Table S8. The Steiger test, as indicated in the additional file (Table S9), supported the conclusion that the causal inference was not influenced by inverse causality.

### 3.3 Sensitivity Analysis

The results of the sensitivity analysis are displayed in Table 2, namely Cochran's Q test, the MR‐Egger intercept test, and the MR‐PRESSO global test. The Cochran Q test indicated heterogeneity in the causality for driving (*Q* = 10.32, *p* = 0.016), but no heterogeneity in causality for the other six phenotypes. Furthermore, there was little evidence of horizontal pleiotropy based on the estimated intercepts from the MR‐Egger regression (Figure 3a,c,e,g). In addition, when systematically discarding individual SNPs, the LOO analysis remained stable (Additional file 2: Figures S1–S7). Moreover, the symmetry of the funnel plot indicated no violation of the estimate, supporting the validity of the findings (Figure 3b,d,f,h).

**TABLE 2:** Sensitivity analysis of the causal association between leisure sedentary behaviours and the risk of GERD.

Exposure	Outcome	Cochran *Q* test		MR‐Egger		MR‐PRESSO *P* value
		*Q* value	*P*	Intercept	*P*	
TV watching	GERD	74.45	0.43	−0.003	0.43	0.80
Computer use	GERD	23.79	0.25	0.010	0.16	0.30
Driving	GERD	10.33	0.02	−0.250	0.20	0.07
Self‐reported MPAs	GERD	9.18	0.06	0.005	0.83	0.14
Self‐reported VPAs	GERD	6.10	0.11	0.030	0.49	0.18
Accelerometer‐measured “average acceleration”	GERD	6.82	0.08	−0.030	0.26	0.17
Accelerometer‐measured fraction accelerations > 425 milligravities	GERD	4.51	0.21	0.030	0.76	0.30

Abbreviations: GERD, gastroesophageal reflux disease; MPAs, moderate physical activities; VPAs, vigorous physical activities.

FIGURE 3Scatter plots and funnel plots from genetically predicted leisure sedentary behaviours and physical activities on gastroesophageal reflux disease. (a, b) Genetically predicted watching TV on gastroesophageal reflux disease (GERD); (c, d) genetically predicted Computer use on GERD; (e, f) genetically predicted self‐reported moderate Physical activity on GERD; (g, h) genetically predicted accelerometer‐measured fraction accelerations > 425 milligravities on GERD.

**Figure:**
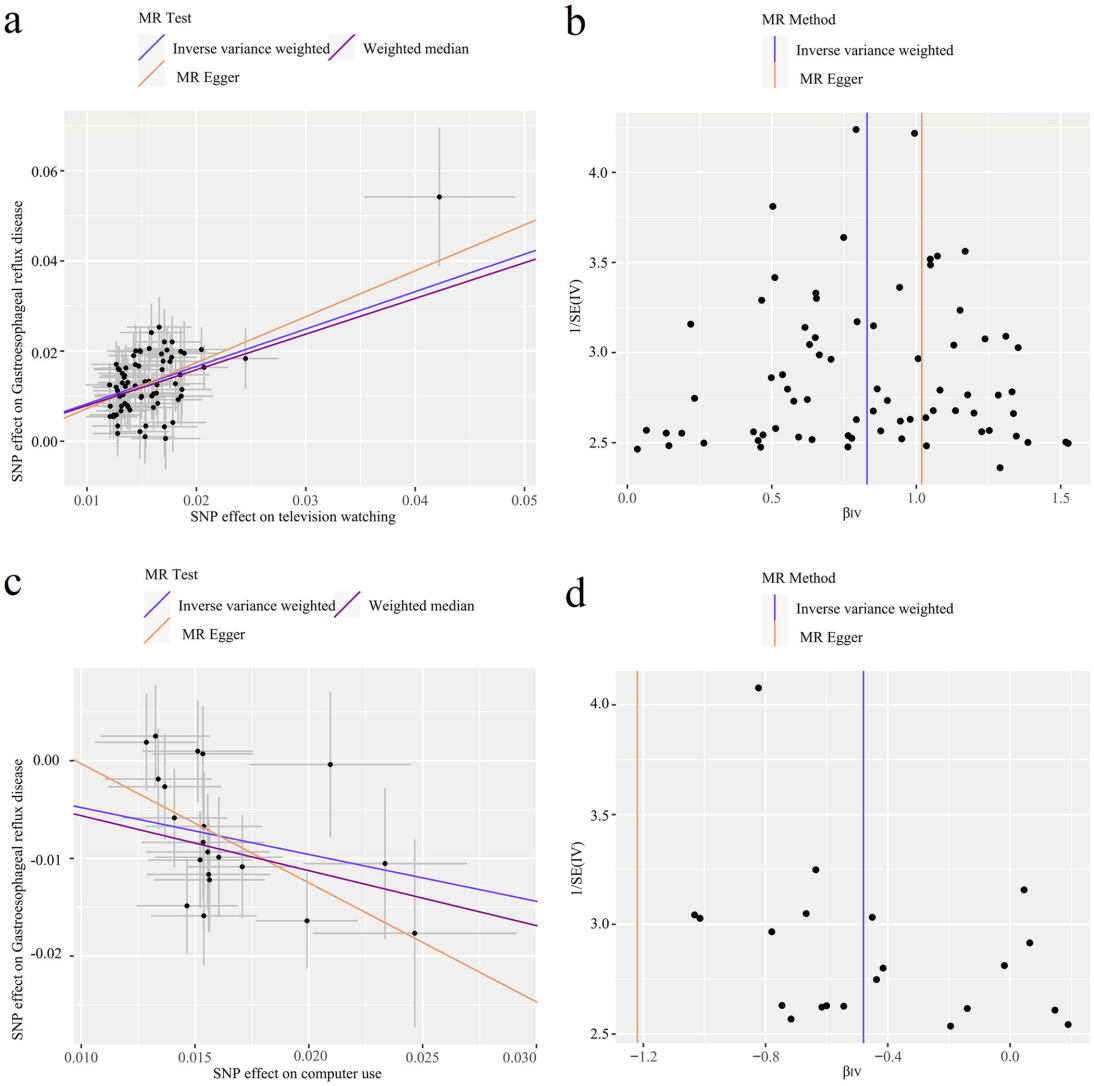

**Figure:**
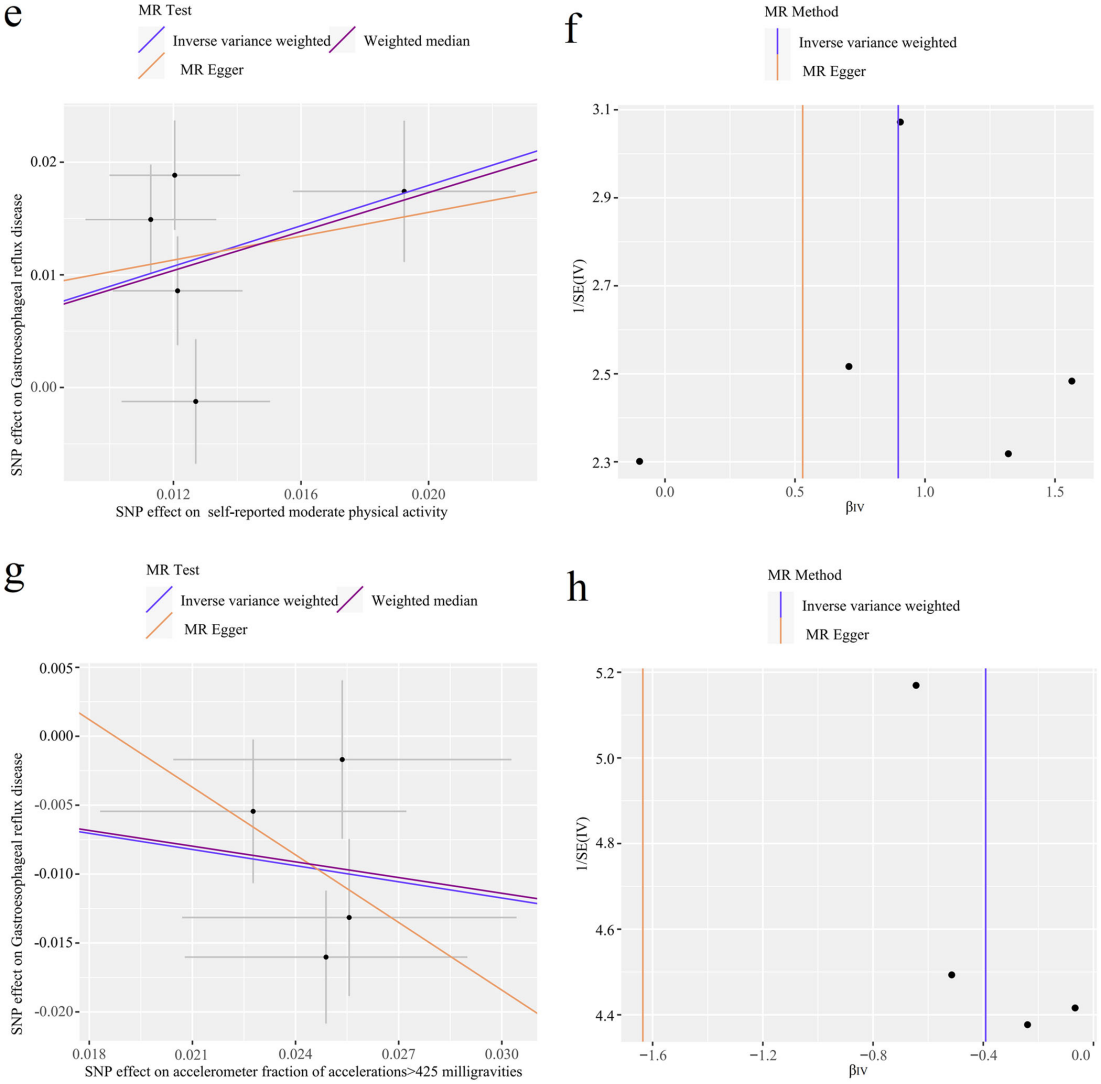

### 3.4 Risk Factor Analysis

To better explore the possible mediating relationship between TV watching, self‐reported MPAs in relation to the increased risk of GERD, MR methods were employed to assess their role in several common risk factors for GERD in this study. Table 3 presents the results of these analyses.

**TABLE 3:** Risk factors analysis.

Exposure	Outcomes	IVW		Heterogeneity		MR‐Egger method	
		Causal effect (95% CI)	*P*	*Q* value	*P*	Intercept	*P*
TV watching	Ever versus never smoked	1.61 (1.35–1.91)	< 0.001	139.93	0.05	0.013	0.99
TV watching	Former versus current smoker	0.72 (0.57–0.90)	0.005	135.6	0.07	0.015	0.09
TV watching	Cigarettes smoked per day	2.11 (0.57–7.80)	0.27	164.74	0.001	−0.025	0.53
TV watching	Total cholesterol	1.13 (1.05–1.21)	0.002	80.99	0.72	0.001	0.08
TV watching	Triglycerides	1.23 (1.15–1.32)	< 0.001	138.00	0.02	−0.005	0.07
TV watching	Body mass index	1.26 (1.19–1.33)	< 0.001	82.75	0.55	−0.005	0.42
TV watching	Type 2 diabetes	2.07 (1.59–2.70)	< 0.001	85.3	0.59	−0.01	0.13
TV watching	Fasting insulin	1.14 (1.09–1.20)	< 0.001	59.84	0.98	0.002	0.53
TV watching	Years of schooling	0.60 (0.53–0.67)	< 0.001	141.14	< 0.001	0.003	0.14
TV watching	Insomnia complaints	1.53 (1.37–1.72)	< 0.001	89.74	> 0.99	−0.001	0.81
TV watching	Visceral adipose tissue volume	1.51 (1.40–1.63)	< 0.001	133.08	0.67	−0.002	0.99
Self‐reported MPAs	Former vs current smoker	0.14 (0.04–0.44)	< 0.001	2.36	0.67	−0.005	0.91
Computer use	Visceral adipose tissue volume	0.83 (0.71–0.97)	0.02	57.13	0.13	0.0001	0.99
Computer use	Years of schooling	1.69 (1.41–2.02)	< 0.001	31.19	0.04	−0.013	0.14

For LSB, an increase of 1 SD hour (equivalent to 2.8 h) spent on TV watching was significantly associated with a 1.13 mmol/L increase in total cholesterol. Additionally, for every 1 SD hour of TV watching, the probability of smoking increased by 1.61 times, while the probability of quitting decreased by 29%. Other associated factors include an increased BMI of 1.26, serum triglyceride levels by 1.23 mmol/L, T2D risk by 2.07 times, fasting insulin by 1.14 pmol/L, insomnia rate by 53%, and visceral adipose tissue volume by 51%.

In the case of self‐reported moderate activity, it was found to be unaffected by increased risk factors for GERD. An additional SD hour spent on computer use was associated with a 29% decrease in visceral adipose tissue volume and 1.69 times higher odds of years of schooling. In contrast, moderate activity was found to increase the probability of smoking cessation.

Overall, the causal effect of MPAs on GERD was found to be independent of the underlying risk factors considered in this study.

## 4 Discussion

This MR study aimed to investigate the causal relationship between LSBs (TV watching and computer use) and PAs (moderate and vigorous) on GERD risk. To achieve this, we employed a robust MR design, including genetic instruments, outcome data, and sensitivity analyses to assess the robustness of the results and detect potential bias or pleiotropy. We utilized multiple MR approaches in a European population. Our findings confirmed that leisure TV watching and self‐reported MPAs were associated with a higher risk of GERD, whereas computer use and accelerometer‐measured fraction accelerations > 425 milligravities are correlated with a lower risk of GERD. However, no causal association was detected between driving, self‐reported VPAs, and accelerometer‐measured “average acceleration” with GERD.

Firstly, our study revealed that self‐reported MPAs may increase the risk of GERD, which is consistent with findings from previous studies. Physical exercise has been associated with various anatomical changes in the gastroesophageal region, such as LES relaxation, oesophageal dilatation, and inhibition of gastroesophageal motor function, thereby increasing the risk of food reflux [13, 37]. However, we observed that an accelerometer‐measured fraction of accelerations > 425 milligravities reduced the risk of GERD. It is believed that physical exercise may reduce the risk of GERD by enhancing the function of the body's anti‐reflux barrier structures, including the diaphragm [38]. Additionally, individuals who engage in more frequent and intense PAs may adopt healthier diets, consuming fewer reflux‐triggering foods such as chocolate and coffee [39]. The discrepancies between self‐reported and accelerometer‐based measurements of PAs in our study can be attributed to differences in measurement methods, such as intensity and standardization [40]. Moreover, accelerometers have limitations in continuously collecting standardized physical data, leading to variability due to measurement deviations (e.g., emotional, cognitive, and social expectations). More importantly, it has been suggested that accelerometer‐measured and self‐reported PAs capture distinct concepts [41], which may explain the discrepancies observed in our study between accelerometer‐based measurements and self‐reported PAs.

Secondly, regarding LBSs, we found that watching TV was associated with a higher risk of GERD, whereas computer use was associated with a lower risk of GERD. Compared to other sedentary leisure behaviours, such as driving and computer use, TV watching is often associated with less rest, shorter durations, lower overall energy expenditure, and increased consumption of snacks, amplifying the negative effects of sedentary leisure behaviour [19]. The longer duration of TV watching may lead to increased exposure to unhealthy food and beverage advertisements, which may promote the desire for high‐calorie and low‐nutrient foods [42, 43]. In contrast to the mentally active state during computer use, watching TV is a recreational activity that reduces self‐reflection and social interaction, potentially leading to consistently poor physical and mental health (including anxiety and depression), which could also contribute to GERD [44, 45]. As for leisure computer use, it may represent individuals engaged in sedentary work who have more stuctured eating patterns, ultimately reducing daytime gastric acid reflux events. Additionally, leisure computer use may be associated with higher education levels. A recent MR analysis found a negative correlation between education level and the occurrence of GERD [46], which could partly explain the protective effect of leisure computer use on GERD.

Furthermore, our risk factor analysis identified several potential explanations for the causal relationship between TV watching and GERD. We found a positive correlation between leisure TV watching and obesity‐related phenotypes (total cholesterol, triglyceride, BMI, visceral adipose tissue volume, T2D, and fasting insulin). Physiological studies have shown that central obesity increases intra‐abdominal and intra‐gastric pressure, raising the gastroesophageal pressure gradient and promoting reflux through mechanical effects [47]. Consistent with this, a meta‐analysis based on data from 18,346 studies reported that being overweight or obese was associated with a 57% and 115% higher risk of GERD, respectively [48]. TV Watching is also associated with an increased likelihood of being a smoker, consistent with multiple studies [49, 50]. In addition, our study revealed that long‐term TV consumption was associated with a reduced probability of smoking cessation. Smoking is considered to be a crucial factor in causing GERD, and further research is needed to explore the mechanism by which smoking behaviour acts as an intermediate factor in the pathway between TV viewing and GERD.

Finally, in our analysis, we found that prolonged TV‐watching time was positively correlated with insomnia, and sleep disturbance was identified as a primary risk factor for GERD in the analysis of unhealthy lifestyles leading to GERD [51]. A prospective study of 2136 adults reported an association between insomnia and the risk of gastroesophageal reflux [52]. The mechanism of oesophageal hyperalgesia confirmed by acid perfusion experiments, caused by sleep deprivation may explain GERD symptoms in individuals with sleep disturbance [53].

Limitations of this study should be acknowledged. Firstly, in our study, part of the GERD diagnoses were based on self‐report, which may affect the credibility of the MR results. Future research should validate our findings when GWAS data on GERD diagnosed solely according to the ICD criteria become available. Secondly, our study only included Europeans, so the results may not be accurately extrapolated to other ethnic groups. Thirdly, exposure and outcome data are partly derived from the same GWAS, which may increase the risk of type 1 errors and bias causal estimation toward detecting a correlation [54]. However, the F statistic >10 dramatically reduces this possibility. With a relatively large sample sizes, the bias caused by sample overlap is expected to be minimal [55]. Additionally, because our study was based on summary statistics, we were unable to perform stratified analyses by age and sex. Furthermore, our study focused on specific LSBs and PAs and did not consider mild PAs and other sedentary behaviours. In the case of the contrasting conclusions derived from the two sedentary leisure behaviours (leisure TV and leisure computer use), although we proposed several hypotheses to explain, there may be uncontrolled biases in the study due to incomplete exclusion of confounding factors. Finally, our study used univariate MR without multiple adjustments for confounding factors such as BMI and obesity, which are known confounders for GERD risk. Our findings indicate that sedentary behaviour and physical activity are associated with GERD. However, the etiology of GERD is multifaceted, involving factors such as dietary habits (e.g., consumption of high‐fat or spicy foods), stress (which can influence gastric acid secretion or lower esophageal sphincter function), medication use (e.g., NSAIDs or calcium channel blockers), and alcohol consumption. Future research should investigate the interactions between these factors to better understand the primary drivers of GERD.

## 5 Conclusion

This MR study provides evidence that public health interventions aimed at reducing sedentary behaviours like excessive TV watching and promoting increased physical activity can potentially impact GERD risk to obtain potential health benefits. Further research in this area, including replication studies and investigations into potential mechanisms, will be valuable in advancing our understanding of the complex relationships between lifestyle factors, metabolic processes, and gastrointestinal health.

## Author Contributions

**Shan Lu:** conceptualization, data curation, writing – original draft, writing – review & editing, investigation. **Yahong Zhu:** methodology, software, data curation, writing – review & editing, investigation. **Mingyu Cui:** data curation, writing – review & editing, investigation. **Zheng Guo:** methodology, writing – review & editing, visualization, validation. **Xingang Li:** conceptualization, writing – original draft, writing – review & editing, supervision. **Ying Song:** supervision, funding acquisition, project administration, writing – original draft, writing – review & editing, investigation.

## Ethics Statement

The current study utilized publicly available summary‐level data from previous genome‐wide association studies (GWAS), which had obtained ethical approval from local institutions and informed consent from respective participants. As our analysis did not involve new data collection or direct interaction with human subjects, separate ethical approval was not applicable.

## Conflicts of Interest

The authors declare no conflicts of interest.

## Transparency Statement

The lead author Xingang Li, Ying Song affirms that this manuscript is an honest, accurate, and transparent account of the study being reported; that no important aspects of the study have been omitted; and that any discrepancies from the study as planned (and, if relevant, registered) have been explained.

## Supporting information

Supporting information.

Supporting information.

## Data Availability

The data that support the findings of this study are available in the IEU open GWAS project at https://gwas.mrcieu.ac.uk/. These data were derived from the following resources available in the public domain: ‐ Ever vs never smoked, https://gwas.mrcieu.ac.uk/datasets/ieu-a-962/ ‐ Former vs current smoker, https://gwas.mrcieu.ac.uk/datasets/ieu-a-963/ ‐ Cigarettes smoked per day, https://gwas.mrcieu.ac.uk/datasets/ieu-a-961/ ‐ Total cholesterol, https://gwas.mrcieu.ac.uk/datasets/ieu-a-301/ ‐ Triglycerides, https://gwas.mrcieu.ac.uk/datasets/ieu-a-302/ ‐ Body mass index, https://gwas.mrcieu.ac.uk/datasets/ieu-a-835/ ‐ Type 2 diabetes, https://gwas.mrcieu.ac.uk/datasets/ieu-a-26/ ‐ Fasting insulin, https://gwas.mrcieu.ac.uk/datasets/ieu-b-115/ ‐ Years of schooling, https://gwas.mrcieu.ac.uk/datasets/ieu-a-755/ ‐ Insomnia complaints, https://gwas.mrcieu.ac.uk/datasets/ebi-a-GCST004695/ ‐ Visceral adipose tissue volume, https://gwas.mrcieu.ac.uk/datasets/ebi-a-GCST90016671/ The data analyzed in the study are available from the corresponding author upon reasonable request.
