# Supplementary material for: Sedentary Leisure Behaviour, Physical Activity, and Gastroesophageal Reflux Disease: Evidence From a Mendelian Randomization Analysis
Source: Health Sci Rep. 2025 Mar 2;8(3):e70479. doi: 10.1002/hsr2.70479 (PMC11872599; doi:10.1002/hsr2.70479)
Supplement: Supplementary file 1 — Supporting information. [file HSR2-8-e70479-s001.docx]

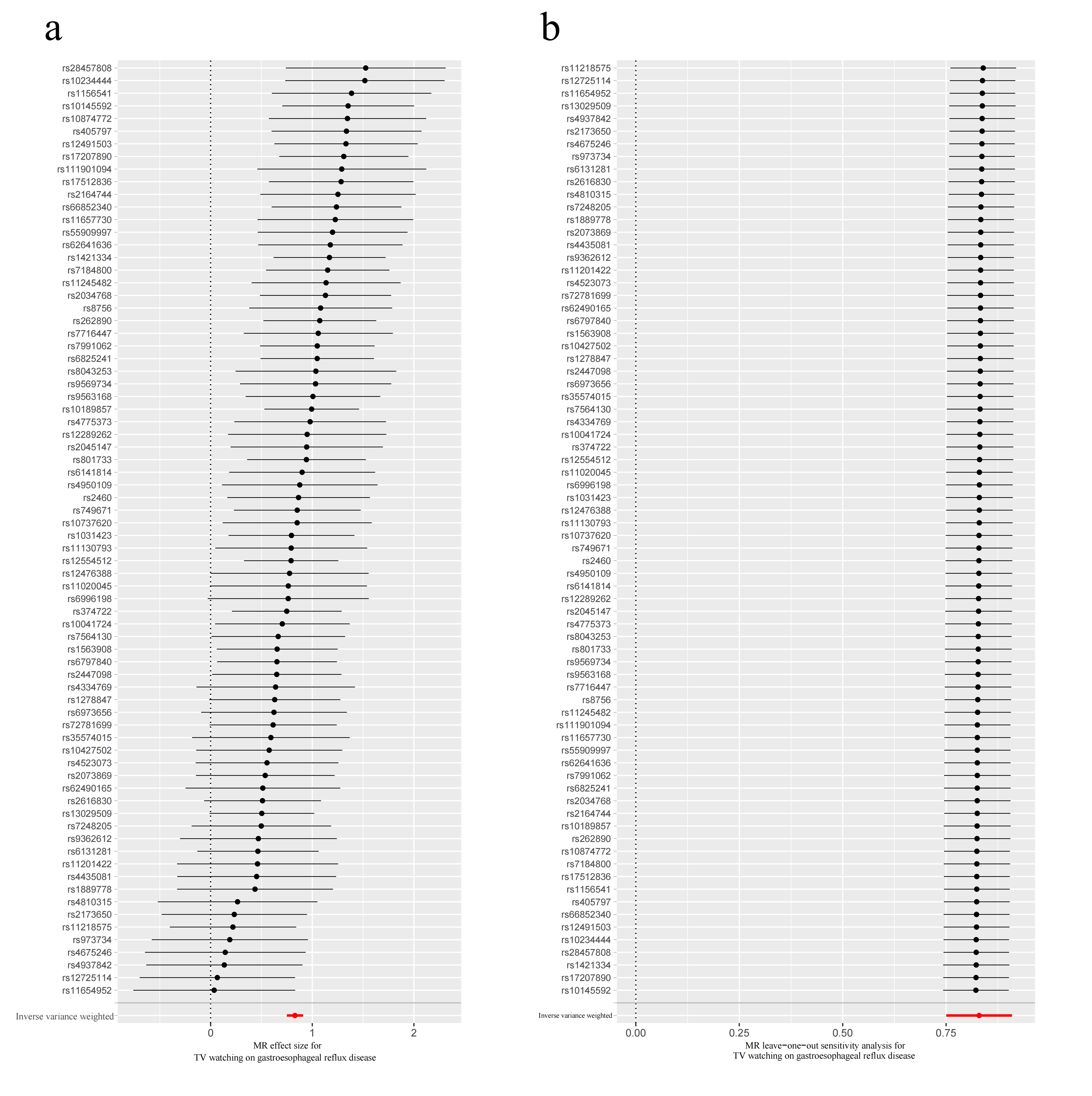


**Fig S1. Forest plot (a) and leave-one-out analysis (b) for TV watching on gastroesophageal reflux disease**.


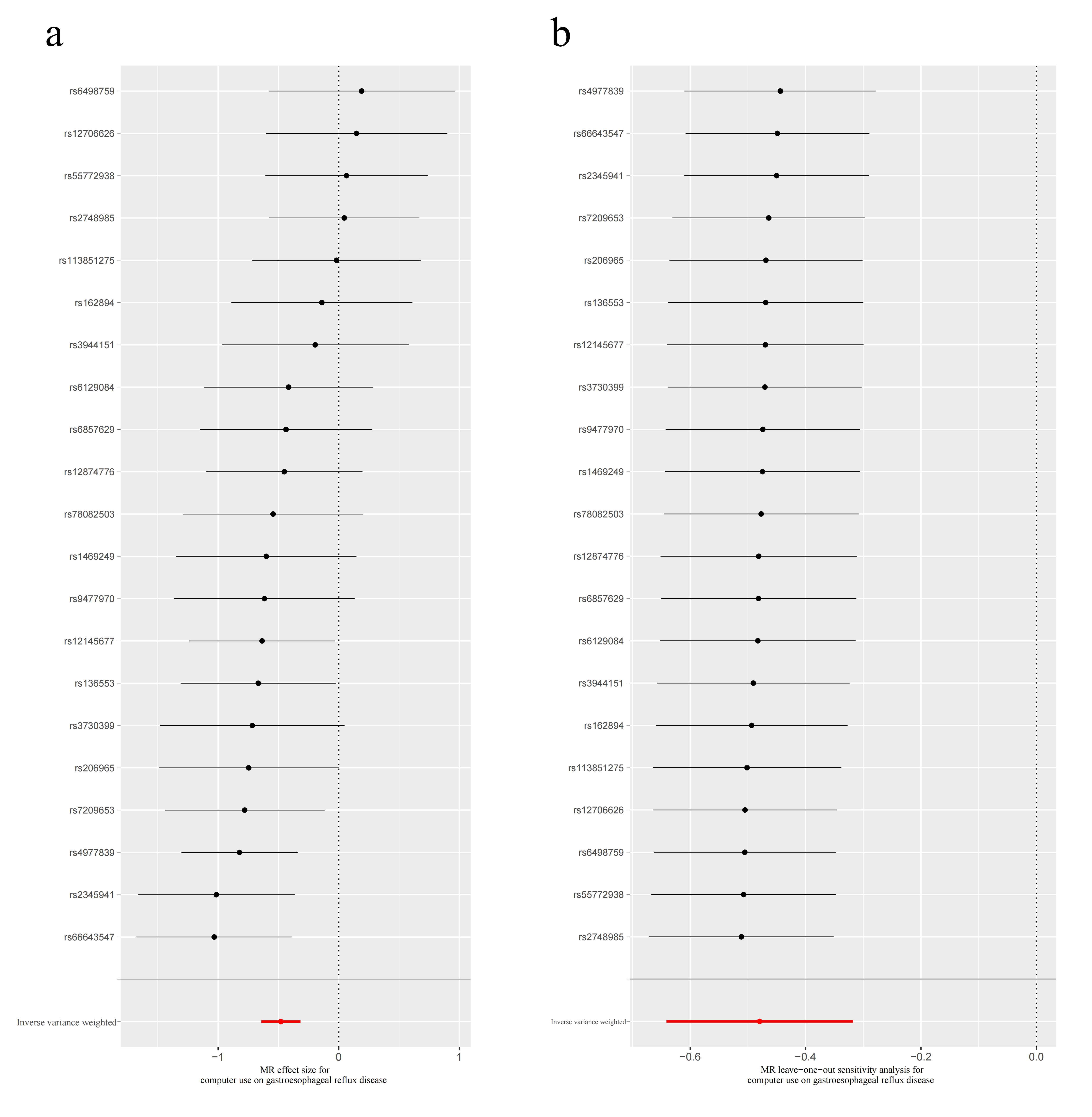


**Fig S2. Forest plot (a) and leave-one-out analysis (b) for computer use on gastroesophageal reflux disease.**


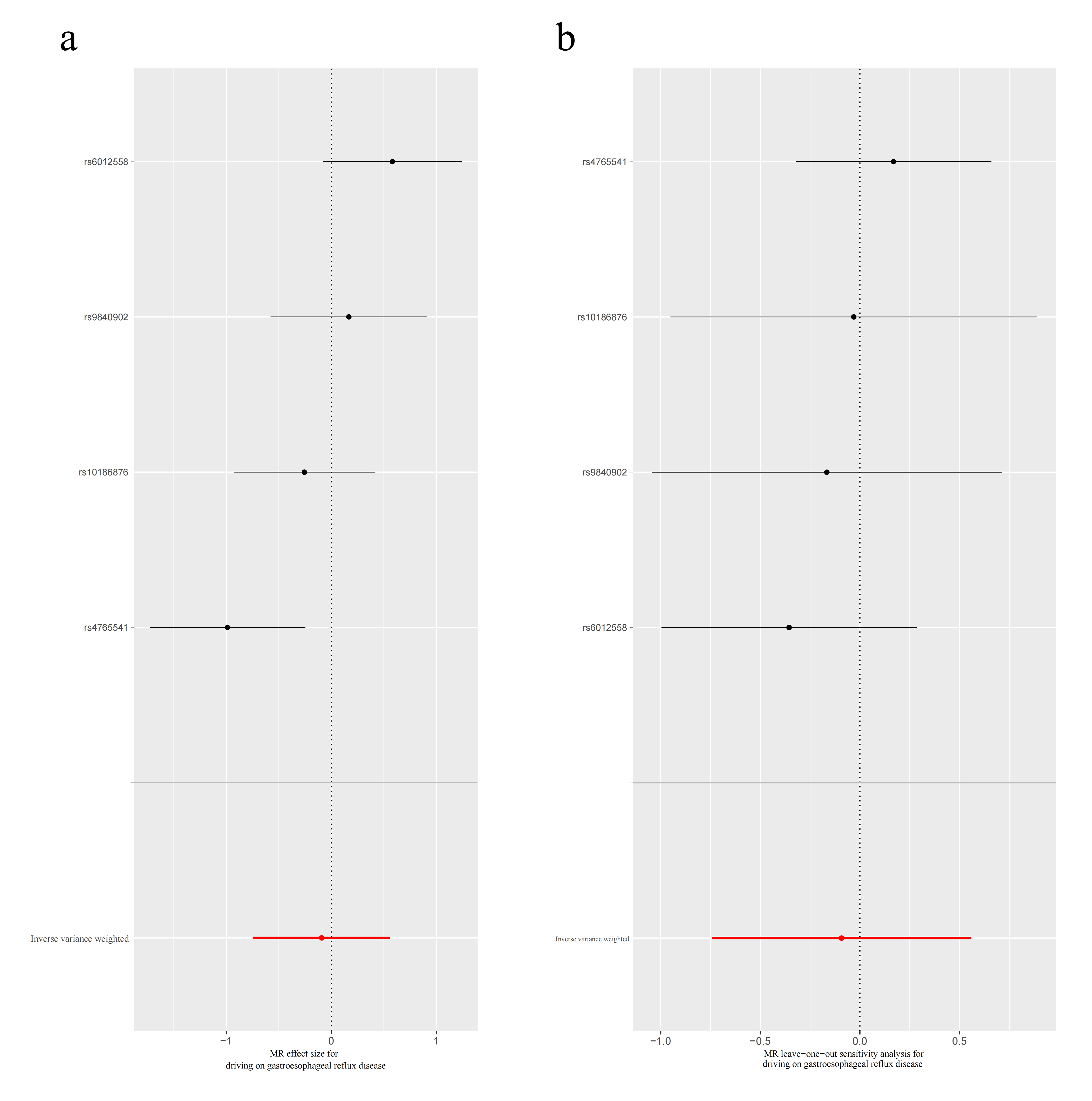


**Fig S3. Forest plot (a) and leave-one-out analysis (b) for driving on gastroesophageal reflux disease.**


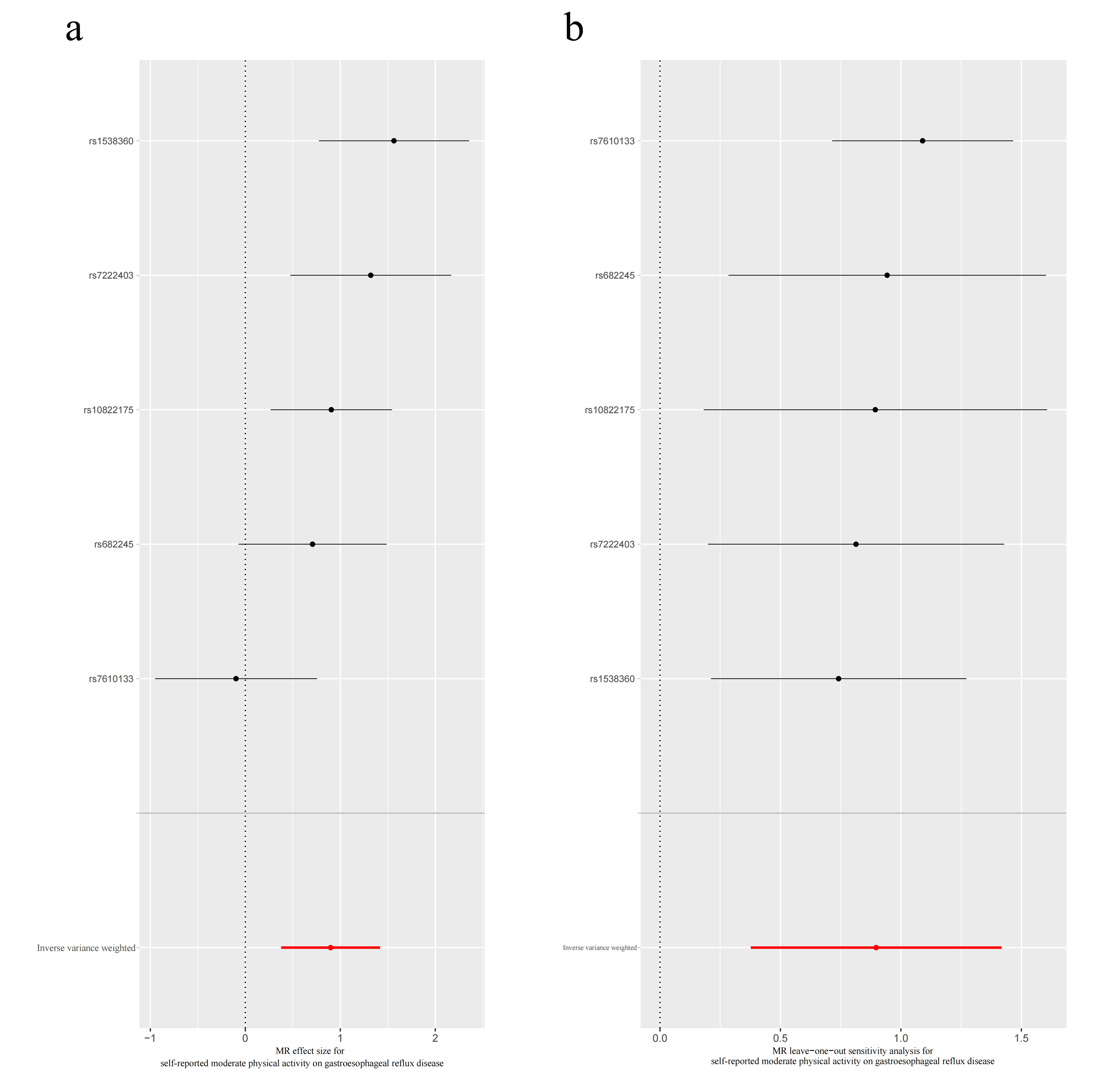


**Fig S4. Forest plot (a) and leave-one-out analysis (b) for self-reported moderate physical activity on gastroesophageal reflux disease.**


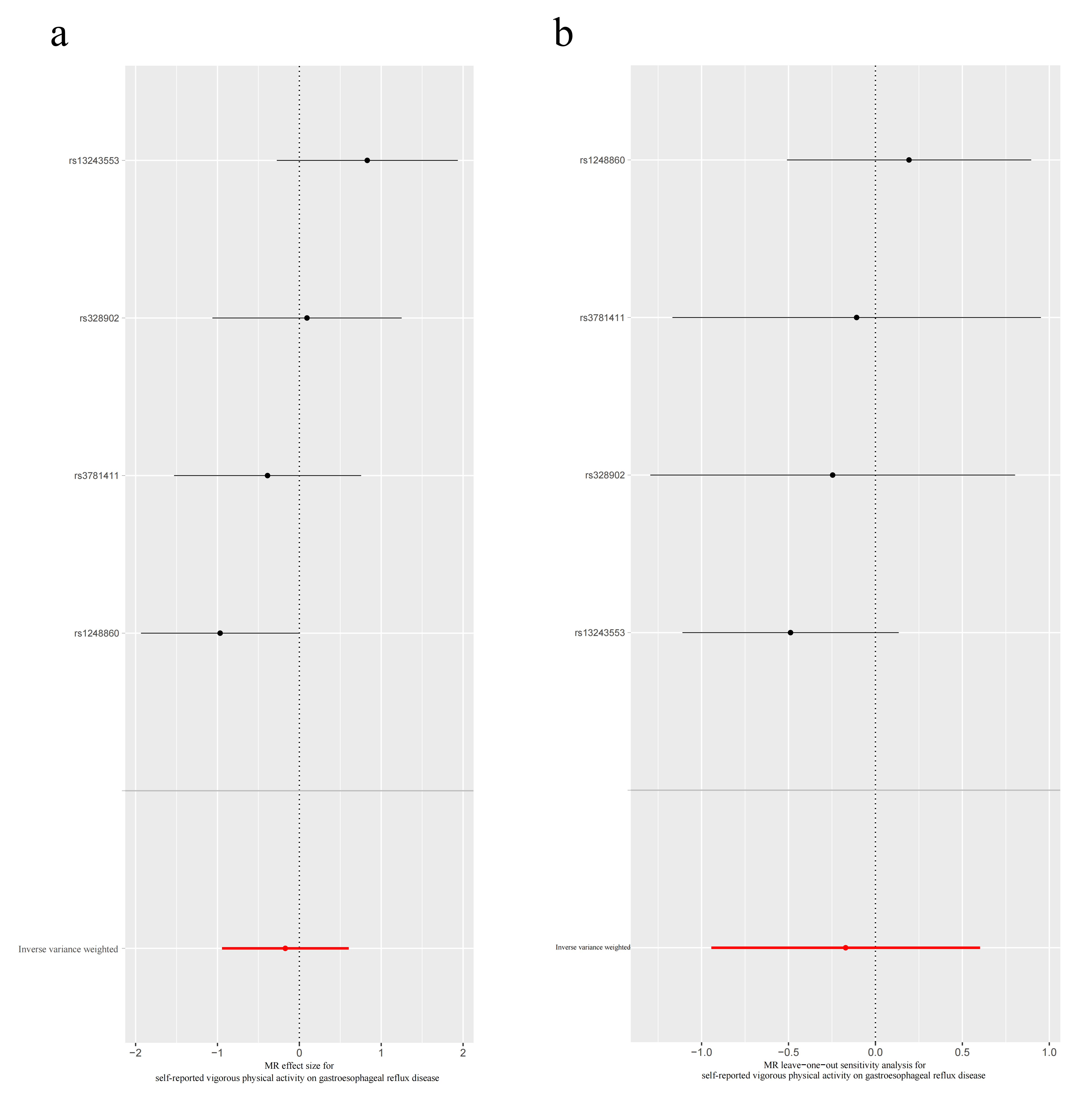


**Fig S5. Forest plot (a) and leave-one-out analysis (b) for self-reported vigorous physical activity on gastroesophageal reflux disease.**


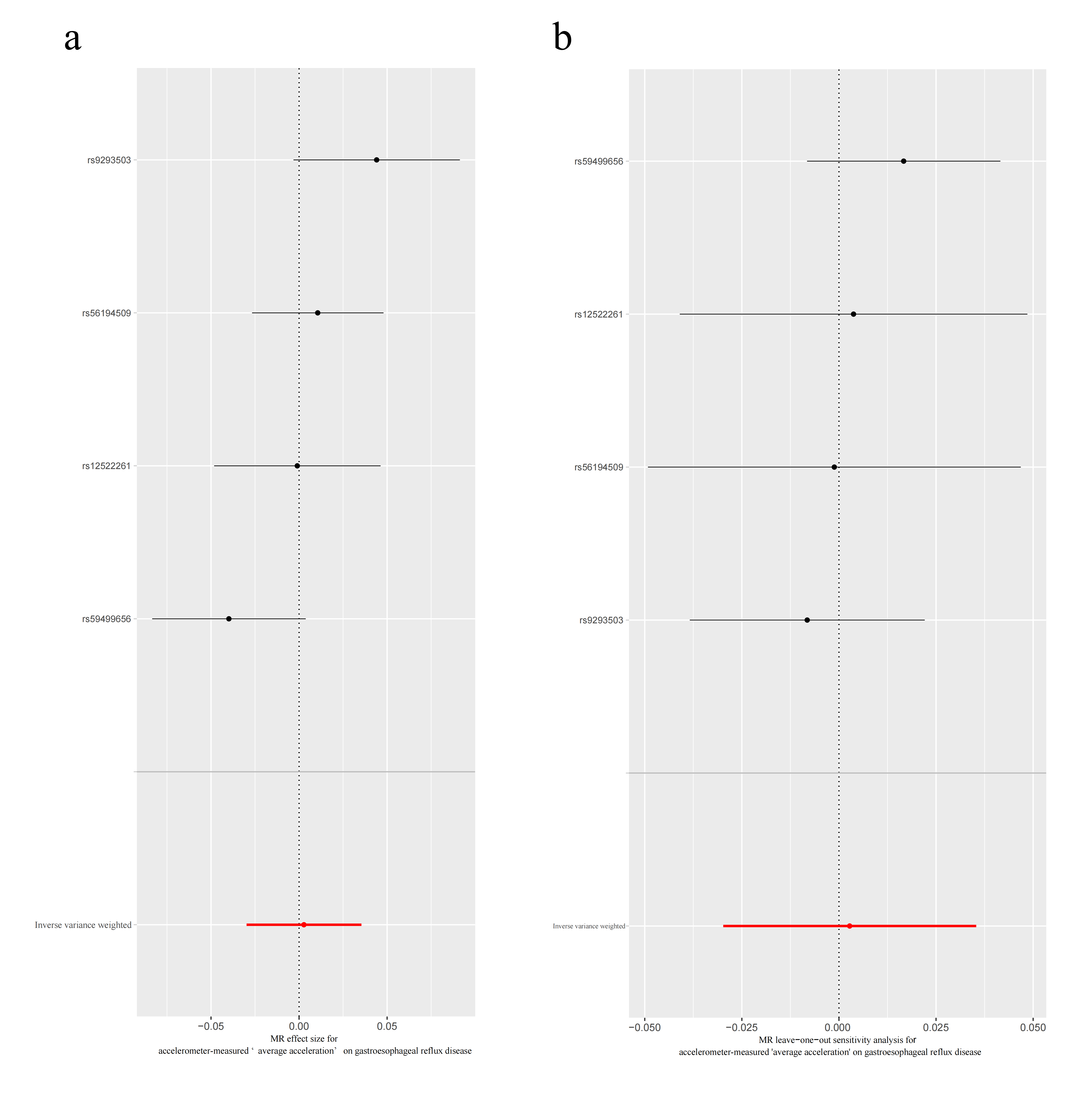


**Fig S6. Forest plot (a) and leave-one-out analysis (b) for accelerometer-measured ‘average acceleration’ on gastroesophageal reflux disease.**


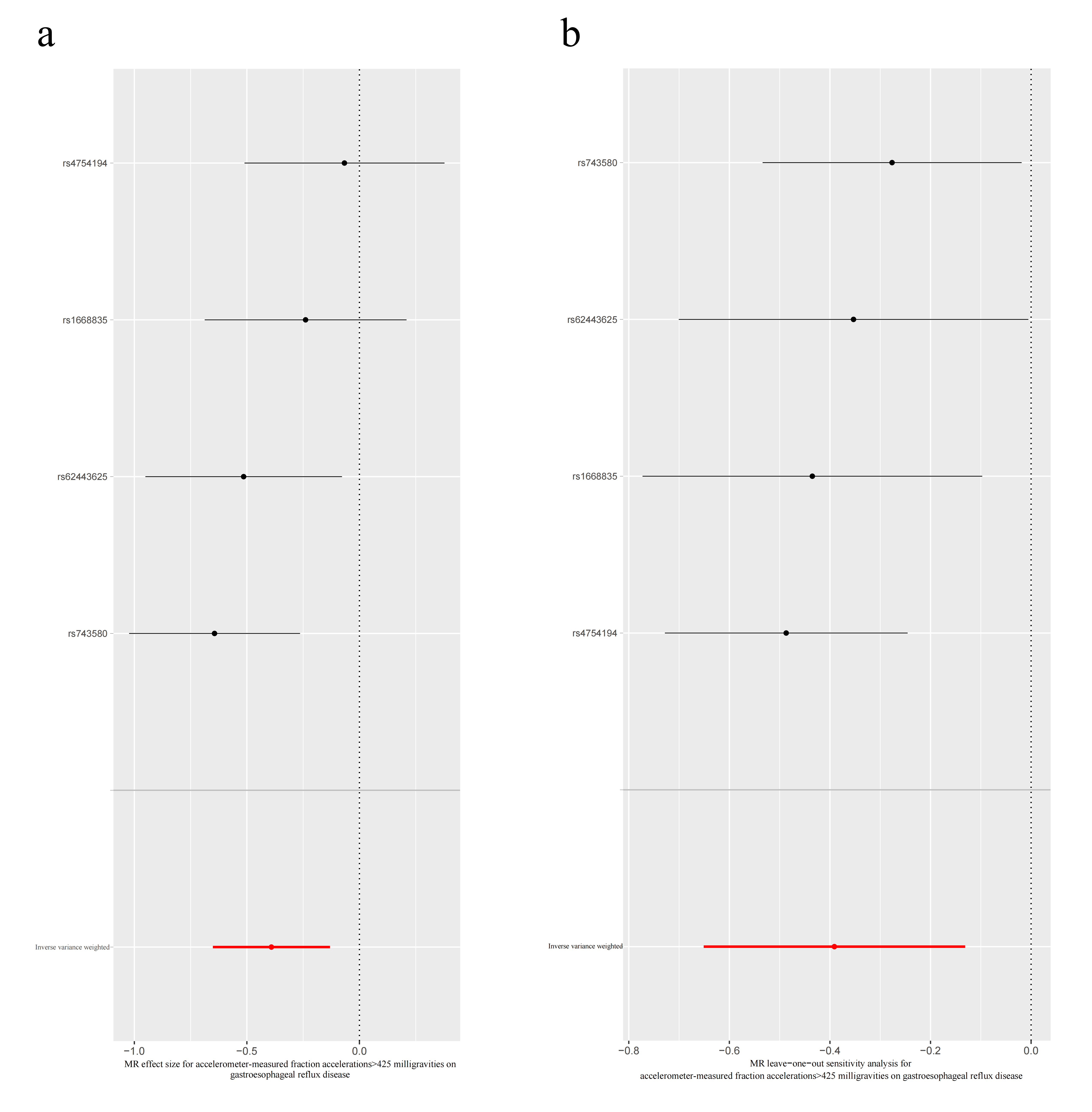


**Fig S7. Forest plot (a) and leave-one-out analysis (b) for accelerometer-measured fraction accelerations > 425 milligravities on gastroesophageal reflux disease.**
