# Supplementary material for: Sedentary Leisure Behaviour, Physical Activity, and Gastroesophageal Reflux Disease: Evidence From a Mendelian Randomization Analysis
Source: Health Sci Rep. 2025 Mar 2;8(3):e70479. doi: 10.1002/hsr2.70479 (PMC11872599; doi:10.1002/hsr2.70479)
Supplement: Supplementary file 2 — Supporting information. [file HSR2-8-e70479-s002.docx]

**Table S1. Instrument variables of TV watching.**

| **SNP** | **Effect allele** | **Other allele** | **Beta** | **SE** | **P** | **R2** | **F** |
| --- | --- | --- | --- | --- | --- | --- | --- |
| rs12554512 | T | C | 0.0207024 | 2.19E-03 | 3.80E-21 | 2.11E-04 | 89.09 |
| rs10189857 | A | G | -0.020463 | 2.18E-03 | 6.20E-21 | 2.09E-04 | 88.11 |
| rs13029509 | G | A | -0.018334 | 2.16E-03 | 2.00E-17 | 1.71E-04 | 72.15 |
| rs374722 | G | A | 0.0244932 | 3.02E-03 | 5.50E-16 | 1.55E-04 | 65.61 |
| rs1421334 | A | C | 0.0173137 | 2.18E-03 | 2.20E-15 | 1.49E-04 | 62.90 |
| rs262890 | A | G | -0.0185683 | 2.36E-03 | 3.20E-15 | 1.47E-04 | 62.13 |
| rs6825241 | C | A | -0.0169583 | 2.17E-03 | 4.90E-15 | 1.45E-04 | 61.29 |
| rs7991062 | C | G | -0.0177381 | 2.29E-03 | 8.80E-15 | 1.42E-04 | 60.14 |
| rs2616830 | G | A | 0.0164651 | 2.17E-03 | 2.90E-14 | 1.37E-04 | 57.81 |
| rs801733 | A | C | 0.0168585 | 2.25E-03 | 7.30E-14 | 1.33E-04 | 55.98 |
| rs6797840 | A | C | -0.0161032 | 2.18E-03 | 1.70E-13 | 1.29E-04 | 54.36 |
| rs1563908 | A | G | 0.0163457 | 2.23E-03 | 2.10E-13 | 1.28E-04 | 53.91 |
| rs6131281 | C | T | 0.0160808 | 2.21E-03 | 3.10E-13 | 1.26E-04 | 53.16 |
| rs7184800 | G | A | 0.0168167 | 2.35E-03 | 8.20E-13 | 1.21E-04 | 51.24 |
| rs11218575 | C | T | 0.0153751 | 2.19E-03 | 2.10E-12 | 1.17E-04 | 49.41 |
| rs1031423 | T | C | -0.0185194 | 2.63E-03 | 1.80E-12 | 1.18E-04 | 49.73 |
| rs749671 | G | A | 0.015654 | 2.24E-03 | 2.70E-12 | 1.16E-04 | 48.92 |
| rs72781699 | G | A | -0.0186804 | 2.68E-03 | 3.00E-12 | 1.15E-04 | 48.66 |
| rs2447098 | C | A | -0.014947 | 2.18E-03 | 6.80E-12 | 1.11E-04 | 47.08 |
| rs17207890 | G | A | 0.015694 | 2.29E-03 | 6.70E-12 | 1.12E-04 | 47.10 |
| rs66852340 | C | T | -0.0177896 | 2.60E-03 | 7.90E-12 | 1.11E-04 | 46.79 |
| rs2034768 | A | G | 0.0147356 | 2.16E-03 | 8.60E-12 | 1.10E-04 | 46.62 |
| rs1278847 | C | A | 0.0159233 | 2.35E-03 | 1.20E-11 | 1.09E-04 | 46.04 |
| rs10145592 | C | G | -0.014845 | 2.21E-03 | 1.80E-11 | 1.07E-04 | 45.20 |
| rs7564130 | T | C | -0.0149864 | 2.25E-03 | 2.80E-11 | 1.05E-04 | 44.34 |
| rs9563168 | G | A | 0.0175868 | 2.67E-03 | 4.30E-11 | 1.03E-04 | 43.47 |
| rs10041724 | T | C | 0.0180931 | 2.74E-03 | 3.90E-11 | 1.03E-04 | 43.65 |
| rs2073869 | C | T | 0.0186112 | 2.91E-03 | 1.50E-10 | 9.70E-05 | 40.97 |
| rs9569734 | A | G | 0.0188846 | 3.01E-03 | 3.40E-10 | 9.34E-05 | 39.44 |
| rs7248205 | C | T | 0.0139253 | 2.22E-03 | 3.40E-10 | 9.34E-05 | 39.46 |
| rs4523073 | A | G | -0.0137517 | 2.21E-03 | 5.00E-10 | 9.16E-05 | 38.68 |
| rs2460 | G | A | -0.0152938 | 2.46E-03 | 5.00E-10 | 9.16E-05 | 38.69 |
| rs8756 | C | A | -0.0134501 | 2.17E-03 | 5.30E-10 | 9.13E-05 | 38.55 |
| rs12491503 | G | A | -0.0142607 | 2.30E-03 | 5.50E-10 | 9.11E-05 | 38.48 |
| rs17512836 | T | C | 0.0422142 | 6.85E-03 | 7.00E-10 | 9.00E-05 | 38.02 |
| rs62641636 | A | G | 0.0144237 | 2.34E-03 | 7.00E-10 | 9.00E-05 | 38.02 |
| rs10427502 | G | A | 0.0136963 | 2.23E-03 | 8.60E-10 | 8.91E-05 | 37.61 |
| rs111901094 | G | T | -0.0170799 | 2.85E-03 | 2.00E-09 | 8.52E-05 | 35.99 |
| rs6973656 | A | G | -0.0134657 | 2.21E-03 | 1.00E-09 | 8.83E-05 | 37.28 |
| rs6141814 | C | A | -0.0135023 | 2.23E-03 | 1.30E-09 | 8.71E-05 | 36.80 |
| rs55909997 | G | A | -0.0135532 | 2.27E-03 | 2.40E-09 | 8.44E-05 | 35.63 |
| rs11245482 | T | C | -0.0132329 | 2.22E-03 | 2.60E-09 | 8.40E-05 | 35.49 |
| rs7716447 | A | G | -0.0133844 | 2.27E-03 | 3.60E-09 | 8.24E-05 | 34.81 |
| rs10737620 | T | A | 0.0144141 | 2.42E-03 | 2.60E-09 | 8.41E-05 | 35.50 |
| rs405797 | T | A | -0.0148849 | 2.51E-03 | 2.80E-09 | 8.36E-05 | 35.29 |
| rs4775373 | T | C | 0.0132229 | 2.26E-03 | 4.70E-09 | 8.13E-05 | 34.32 |
| rs2173650 | G | T | 0.017845 | 3.04E-03 | 4.60E-09 | 8.14E-05 | 34.37 |
| rs11130793 | C | T | 0.0129223 | 2.21E-03 | 5.00E-09 | 8.09E-05 | 34.17 |
| rs2045147 | A | G | 0.0126686 | 2.18E-03 | 5.90E-09 | 8.02E-05 | 33.88 |
| rs11657730 | C | T | 0.0129477 | 2.26E-03 | 1.10E-08 | 7.74E-05 | 32.68 |
| rs4950109 | T | C | -0.012789 | 2.22E-03 | 9.00E-09 | 7.82E-05 | 33.04 |
| rs1889778 | C | T | 0.0124324 | 2.17E-03 | 9.60E-09 | 7.80E-05 | 32.92 |
| rs2164744 | T | C | -0.0128573 | 2.25E-03 | 1.10E-08 | 7.75E-05 | 32.75 |
| rs62490165 | A | T | -0.0131108 | 2.29E-03 | 1.00E-08 | 7.76E-05 | 32.75 |
| rs4937842 | G | C | -0.0128044 | 2.26E-03 | 1.40E-08 | 7.63E-05 | 32.20 |
| rs973734 | C | A | 0.0170761 | 3.01E-03 | 1.50E-08 | 7.60E-05 | 32.11 |
| rs9362612 | T | A | 0.0124319 | 2.20E-03 | 1.50E-08 | 7.60E-05 | 32.07 |
| rs28457808 | C | G | 0.0165953 | 2.93E-03 | 1.50E-08 | 7.58E-05 | 31.99 |
| rs11020045 | A | C | -0.0129952 | 2.30E-03 | 1.60E-08 | 7.55E-05 | 31.88 |
| rs12725114 | G | A | 0.015309 | 2.71E-03 | 1.60E-08 | 7.55E-05 | 31.88 |
| rs12289262 | C | T | -0.0137276 | 2.45E-03 | 2.00E-08 | 7.46E-05 | 31.51 |
| rs10234444 | G | A | 0.0158909 | 2.86E-03 | 2.90E-08 | 7.29E-05 | 30.77 |
| rs4334769 | G | T | 0.0121315 | 2.17E-03 | 2.10E-08 | 7.43E-05 | 31.36 |
| rs10874772 | G | A | 0.0126769 | 2.26E-03 | 1.90E-08 | 7.47E-05 | 31.54 |
| rs12476388 | C | T | 0.0133031 | 2.37E-03 | 2.10E-08 | 7.44E-05 | 31.40 |
| rs4435081 | C | T | 0.0121101 | 2.17E-03 | 2.30E-08 | 7.39E-05 | 31.22 |
| rs4810315 | G | A | -0.0128121 | 2.30E-03 | 2.70E-08 | 7.32E-05 | 30.90 |
| rs1156541 | C | T | 0.0144511 | 2.60E-03 | 2.80E-08 | 7.31E-05 | 30.87 |
| rs35574015 | T | C | -0.0131592 | 2.38E-03 | 3.00E-08 | 7.27E-05 | 30.69 |
| rs4675246 | G | T | -0.0148496 | 2.68E-03 | 2.90E-08 | 7.29E-05 | 30.80 |
| rs11201422 | T | C | 0.0126882 | 2.30E-03 | 3.40E-08 | 7.21E-05 | 30.46 |
| rs8043253 | C | T | -0.0120657 | 2.19E-03 | 3.40E-08 | 7.21E-05 | 30.45 |
| rs6996198 | C | T | -0.0163982 | 2.97E-03 | 3.50E-08 | 7.20E-05 | 30.41 |
| rs11654952 | T | G | -0.0171839 | 3.14E-03 | 4.30E-08 | 7.11E-05 | 30.03 |

**Table S2. Instrument variables of computer use.**

| **SNP** | **Effect allele** | **Other allele** | **Beta** | **SE** | **P** | **R^2^** | **F** |
| --- | --- | --- | --- | --- | --- | --- | --- |
| rs4977839 | G | A | -0.0199181 | 2.23E-03 | 4.70E-19 | 1.88E-04 | 79.56 |
| rs66643547 | C | T | -0.0153853 | 2.32E-03 | 3.20E-11 | 1.04E-04 | 44.05 |
| rs2345941 | A | G | 0.014647 | 2.22E-03 | 4.00E-11 | 1.03E-04 | 43.64 |
| rs7209653 | T | C | 0.0156381 | 2.41E-03 | 9.10E-11 | 9.95E-05 | 42.01 |
| rs12145677 | G | A | -0.0170774 | 2.41E-03 | 1.20E-12 | 1.19E-04 | 50.42 |
| rs136553 | C | T | -0.0152252 | 2.28E-03 | 2.30E-11 | 1.06E-04 | 44.69 |
| rs206965 | T | C | 0.0156007 | 2.71E-03 | 9.10E-09 | 7.82E-05 | 33.02 |
| rs3730399 | A | G | 0.0246511 | 4.46E-03 | 3.30E-08 | 7.23E-05 | 30.53 |
| rs9477970 | T | A | -0.0160441 | 2.80E-03 | 1.00E-08 | 7.77E-05 | 32.82 |
| rs1469249 | G | A | 0.0155576 | 2.71E-03 | 9.50E-09 | 7.80E-05 | 32.94 |
| rs78082503 | G | C | -0.0153586 | 2.69E-03 | 1.10E-08 | 7.73E-05 | 32.66 |
| rs12874776 | C | A | -0.0233491 | 3.59E-03 | 8.00E-11 | 1.00E-04 | 42.26 |
| rs6857629 | G | A | 0.0153859 | 2.56E-03 | 1.90E-09 | 8.55E-05 | 36.12 |
| rs6129084 | A | T | -0.0140927 | 2.30E-03 | 9.30E-10 | 8.87E-05 | 37.46 |
| rs3944151 | A | G | 0.0136733 | 2.47E-03 | 3.10E-08 | 7.26E-05 | 30.66 |
| rs6498759 | T | C | 0.0132648 | 2.39E-03 | 2.70E-08 | 7.32E-05 | 30.90 |
| rs12706626 | G | A | -0.012866 | 2.27E-03 | 1.40E-08 | 7.63E-05 | 32.22 |
| rs162894 | T | G | 0.0133847 | 2.34E-03 | 1.10E-08 | 7.75E-05 | 32.71 |
| rs55772938 | A | G | -0.0151223 | 2.42E-03 | 3.90E-10 | 9.27E-05 | 39.14 |
| rs2748985 | T | C | -0.0153444 | 2.21E-03 | 4.10E-12 | 1.14E-04 | 48.09 |
| rs113851275 | G | A | -0.0209442 | 3.54E-03 | 3.20E-09 | 8.30E-05 | 35.06 |

**Table S3. Instrument variables of driving.**

| **SNP** | **Effect allele** | **Other allele** | **Beta** | **SE** | **P** | **R^2^** | **F** |
| --- | --- | --- | --- | --- | --- | --- | --- |
| rs10186876 | A | G | 0.0144048 | 2.34E-03 | 7.20E-10 | 8.99E-05 | 37.96 |
| rs4765541 | T | C | 0.0137597 | 2.36E-03 | 5.10E-09 | 8.08E-05 | 34.13 |
| rs6012558 | G | A | 0.0144779 | 2.26E-03 | 1.60E-10 | 9.69E-05 | 40.90 |
| rs9840902 | G | A | -0.0148575 | 2.72E-03 | 4.50E-08 | 7.08E-05 | 29.92 |

**Table S4.** **Instrument variables of self-reported moderate physical activity.**

| **SNP** | **Effect allele** | **Other allele** | **Beta** | **SE** | **P** | **R^2^** | **F** |
| --- | --- | --- | --- | --- | --- | --- | --- |
| rs10822175 | T | A | -0.019235 | 3.49E-03 | 3.50E-08 | 8.84E-05 | 30.39 |
| rs1538360 | G | A | -0.0120408 | 2.04E-03 | 3.80E-09 | 1.01E-04 | 34.73 |
| rs682245 | C | T | 0.0121307 | 2.03E-03 | 2.30E-09 | 1.04E-04 | 35.74 |
| rs7222403 | C | T | 0.0112894 | 2.05E-03 | 3.60E-08 | 8.83E-05 | 30.38 |
| rs7610133 | C | T | -0.0126995 | 2.33E-03 | 4.70E-08 | 8.67E-05 | 29.82 |

**Table S5.** **Instrument variables of self-reported vigorous physical activity.**

| **SNP** | **Effect allele** | **Other allele** | **Beta** | **SE** | **P** | **R^2^** | **F** |
| --- | --- | --- | --- | --- | --- | --- | --- |
| rs1248860 | A | G | 0.00976972 | 1.31E-03 | 1.10E-13 | 2.12E-04 | 55.26 |
| rs13243553 | A | G | -0.00874851 | 1.35E-03 | 9.00E-11 | 1.61E-04 | 42.02 |
| rs328902 | T | C | 0.00878858 | 1.42E-03 | 5.50E-10 | 1.47E-04 | 38.48 |
| rs3781411 | T | C | -0.0125792 | 2.00E-03 | 3.00E-10 | 1.52E-04 | 39.67 |

**Table S6.** **Instrument variables of accelerometer-measured ‘average acceleration’.**

| **SNP** | **Effect allele** | **Other allele** | **Beta** | **SE** | **P** | **R^2^** | **F** |
| --- | --- | --- | --- | --- | --- | --- | --- |
| rs12522261 | A | G | -0.210522 | 3.83E-02 | 3.90E-08 | 3.32E-04 | 30.21 |
| rs56194509 | G | T | 0.303404 | 4.39E-02 | 5.00E-12 | 5.23E-04 | 47.68 |
| rs59499656 | T | A | 0.228253 | 3.83E-02 | 2.40E-09 | 3.91E-04 | 35.60 |
| rs9293503 | C | T | -0.328953 | 5.87E-02 | 2.10E-08 | 3.45E-04 | 31.42 |

**Table S7.** **Instrument variables of accelerometer-measured fraction accelerations > 425 milli-gravities.**

| **SNP** | **Effect allele** | **Other allele** | **Beta** | **SE** | **P** | **R^2^** | **F** |
| --- | --- | --- | --- | --- | --- | --- | --- |
| rs1668835 | T | A | -0.022767 | 4.45E-03 | 3.10E-07 | 2.89E-04 | 26.18 |
| rs4754194 | C | T | -0.0253582 | 4.91E-03 | 2.40E-07 | 2.94E-04 | 26.64 |
| rs62443625 | T | C | -0.0255601 | 4.86E-03 | 1.40E-07 | 3.05E-04 | 27.68 |
| rs743580 | A | G | 0.024886 | 4.10E-03 | 1.30E-09 | 4.05E-04 | 36.76 |

**Table S8. MR estimates of the causal association between leisure sedentary behaviours and physical activity and the risk of gastroesophageal reflux disease.**

| **Exposure** | **Methods** | **Gastroesophageal reflux disease** | |
| --- | --- | --- | --- |
|  |  | **OR (95% CI)** | **P** |
| TV watching | IVW | 2.29 (2.12-2.48) | 1.75E-93 |
|  | WM | 2.21 (1.95-2.49) | 1.75E-36 |
|  | MR-Egger | 2.77 (1.72-4.45) | 7.15E-05 |
| Computer use | IVW | 0.62 (0.53-0.73) | 5.67E-09 |
|  | WM | 0.57 (0.46-0.70) | 7.90E-07 |
|  | MR-Egger | 0.30 (0.11-0.81) | 2.86E-02 |
| Driving | IVW | 0.91 (0.48-1.75) | 7.80E-01 |
|  | WM | 0.99 (0.62-1.60) | 1.00E+00 |
|  | MR-Egger | 3.20E07(0.42-2.42E+15) | 2.00E-01 |
| Self-reported moderate physical activity | IVW | 2.45 (1.46-4.13) | 7.00E-04 |
|  | WM | 2.38 (1.42-3.97) | 7.00E-04 |
|  | MR-Egger | 1.70 (0.08-38.27) | 7.60E-01 |
| Self-reported vigorous physical activity | IVW | 0.84 (0.39-1.82) | 6.60E-01 |
|  | WM | 0.80 (0.40-1.62) | 5.30E-01 |
|  | MR-Egger | 0.06 (0.0001-31.74) | 4.70E-01 |
| Accelerometer-measured ‘average acceleration’ | IVW | 1.00 (0.97-1.04) | 8.70E-01 |
|  | WM | 1.01(0.98-1.03) | 7.10E-01 |
|  | MR-Egger | 1.13 (0.97-1.31) | 2.60E-01 |
| Accelerometer-measured fraction accelerations > 425 milli-gravities | IVW | 0.68 (0.52-0.88) | 3.00E-03 |
|  | WM | 0.68 (0.52-0.89) | 5.00E-03 |
|  | MR-Egger | 0.19 (0.0002-183.77) | 6.90E-01 |

**Table S9. Steiger text.**

| **Exposure** | **Methods** | **Gastroesophageal reflux disease** | |
| --- | --- | --- | --- |
|  |  | Correct causal direction | Steiger **P** |
| TV watching | Steiger test | TRUE | 4.47E-195 |
| Computer use | Steiger test | TRUE | 1.25E-64 |
| Driving | Steiger test | TRUE | 1.44E-12 |
| Self-reported moderate physical activity | Steiger test | TRUE | 6.18E-11 |
| Self-reported vigorous physical activity | Steiger test | TRUE | 4.32E-22 |
| Accelerometer-measured ‘average acceleration’ | Steiger test | TRUE | 9.26E-25 |
| Accelerometer-measured fraction accelerations > 425 milli-gravities | Steiger test | TRUE | 8.84E-18 |
